# High Doses of *Bupleurum falcatum* Partially Prevents Estrogen Deficiency-Induced Bone Loss With Anti-osteoclastogenic Activity Due to Enhanced iNOS/NO Signaling

**DOI:** 10.3389/fphar.2018.01314

**Published:** 2018-11-16

**Authors:** Mijung Yeom, Eun-Young Kim, Jae-Hyun Kim, Hyuk-Sang Jung, Youngjoo Sohn

**Affiliations:** ^1^Acupuncture and Meridian Science Research Center, College of Korean Medicine, Kyung Hee University, Seoul, South Korea; ^2^Department of Anatomy, College of Korean Medicine, Kyung Hee University, Seoul, South Korea

**Keywords:** *Bupleurum falcatum*, postmenopausal osteoporosis, bone resorption, osteoclasts, ovariectomized rats

## Abstract

**Background and Objective:** *Bupleurum falcatum* (BF) extract, a natural product with anti-inflammatory properties, has been traditionally used to treat menopausal symptoms, but its role in osteoporosis, another serious health concern of menopausal women, remains unknown. Here we investigated whether and how BF prevents estrogen deficiency-induced bone loss using both *in vitro* and *in vivo* models.

**Methods:** Female Sprague-Dawley rats were ovariectomized (OVX) and subjected to oral BF treatment daily for 8 weeks. Additionally, pre-osteoclastic RAW 264.7 cells were employed to evaluate the effects of BF and its underlying mechanism on receptor activator of nuclear factor kappa-B ligand (RANKL)-induced osteoclast formation *in vitro*.

**Results:** A high dose of BF partially prevented ovariectomy (OVX)-induced bone loss and reduced the levels of tartrate-resistant acid phosphatase (TRAP) in serum and osteoclast numbers in femurs of OVX rats. Furthermore, BF clearly inhibited RANKL-induced osteoclast differentiation and bone resorption activity in RAW 264.7 cells. BF also inhibited the osteoclastogenic transcription factors c-Fos and nuclear factor of activated T cells c1 (NFATc1) and, consequently, downregulated the expression of osteoclast marker genes. Moreover, BF upregulated interferon-β (IFN-β)/inducible nitric oxide synthase (iNOS)/nitric oxide (NO) signaling, even though it had no impact on mitogen-activated protein kinases (MAPK) or NF-κB. The inhibition of osteoclast formation by BF was abrogated by iNOS-specific inhibitors. Consistent with cellular studies, BF upregulated iNOS protein expression in femurs from OVX rats.

**Conclusion:** Taken together, our results indicate that BF partially prevented estrogen deficiency-induced bone loss with anti-osteoclastogenic activity potentially due to enhanced iNOS/NO signaling.

## Introduction

Osteoporosis, an emerging global health issue, is a skeletal disorder associated with low bone mass leading to bone fragility and increased risk of fracture. It is prevalent in the elderly, particularly in postmenopausal women (Wark, 1996; Black and Rosen, 2016). Bone mass is regulated through bone remodeling, which consists of bone resorption followed by new bone formation. Normally, bone formation and resorption are tightly coordinated during the remodeling cycle, but when this coordination is lost in certain pathological conditions such as lower estrogen levels during menopause and postmenopause, abnormal bone remodeling, mainly leading to bone loss, ensues (Feng and McDonald, 2011). The increased pro-inflammatory cytokines that occur in estrogen deficiency as well as chronic inflammation contributes to bone loss by promoting osteoclast differentiation (Brincat et al., 2014; Amarasekara et al., 2015).

A common feature of bone loss is excessive activity of bone-resorbing cells, known as osteoclasts (OCs). OCs are highly specialized multinucleated giant cells derived from the monocyte/macrophage lineage. During the process of osteoclastogenesis, OC progenitors differentiate into mononuclear pre-OCs, which then fuse to form mature multinucleated OCs that have bone resorption activity (Teitelbaum and Ross, 2003). OC differentiation and activity are regulated by receptor activator of nuclear factor kappa-B (NF-κB) ligand (RANKL), the master regulator of osteoclastogenesis (Boyle et al., 2003). The binding of RANKL to its receptor, RANK, stimulates the NF-κB and mitogen-activated protein kinase (MAPK) pathways by subsequently interacting with Tumor necrosis factor receptor- associated factor 6 (TRAF6), which in turn induces the expression of the master osteoclastogenic transcription factors, nuclear factor of activated T cells c1 (NFATc1) and osteosarcoma oncogene c-Fos, thereby increasing the expression of several genes typically expressed by OCs, such as cathepsin K, tartrate-resistant acid phosphatase (TRAP), calcitonin receptor, and metalloproteinase (MMP)-9 (Asagiri and Takayanagi, 2007; Takayanagi, 2007a). RANKL-dependent OC differentiation can also be controlled via negative feedback. A typical negative regulator of RANKL signaling is interferon beta (IFN-β), which is produced by RANKL via c-Fos, in turn attenuating excessive RANKL signaling by inhibiting RANKL-induced c-Fos expression (Takayanagi et al., 2002). Furthermore, inducible nitric oxide synthase (iNOS) and nitric oxide (NO) have recently been demonstrated to be involved in negative feedback regulation pertinent to the anti-resorptive effects of IFN-β (Abraham et al., 2009).

Although several agents, including estrogens, and bisphosphonates, are currently available for the prevention and treatment of postmenopausal osteoporosis, various side effects of these anti-resorptive agents lead many patients to discontinue their use (Khan et al., 2014; Tella and Gallagher, 2014; Herrera et al., 2015). Given the limitations of current options for managing postmenopausal osteoporosis, there is an unmet need for alternatives with minimal adverse effects.

The medicinal plant *Bupleurum falcatum* (BF; family *Apiaceae*) is a popular herbal medicine used throughout East Asia, including Korea. Traditionally, its dried roots (“Shiho” in Korean), the part used for medicinal purposes, have been widely used for treating the common cold, fever, hepatitis, inflammation, and also the symptoms of menopausal syndrome, such as hot flashes and depressive mood change (Yang and Wang, 2006; Yang et al., 2017). Indeed, several *in vitro, in vivo*, and clinical studies have demonstrated that BF crude extracts or its active components such as saikosaponins have potential anti-inflammatory, antipyretic, hepatoprotective, neuroprotective, and immunomodulatory effects (Yang et al., 2017). In a clinical trial, a decoction including BF as a major ingredient showed overall efficacy for relieving both the vasomotor and psychological symptoms of postmenopausal women with climacteric symptoms (Hidaka et al., 2013). In an *in vivo* study, BF was reported to have potent hypothermic and antipyretic effects (Takagi and Shibata, 1969). Another *in vivo* study showed that BF had antidepressant-like activity in the tail suspension test (Kwon et al., 2010). Based on its anti-inflammatory properties as well as beneficial effects on menopausal symptoms, we thus hypothesized that BF may also have preventive effects against menopause-related bone loss.

In the present study, we examined the protective effects of BF in menopause-related bone loss *in vivo* using ovariectomized (OVX) rats which exhibit estrogen deficiency, and elucidated its cellular and molecular mechanisms of action in pre-osteoclastic RAW 264.7 cells which are widely used for the study of osteoclastogenesis *in vitro* (Collin-Osdoby and Osdoby, 2012).

## Materials and Methods

### Preparation of BF Extract

Dried roots of *B*. *falcatum* L were purchased from Kyung Hee University Medical Center and authenticated by Professor Yungmin Bu at the Laboratory of Herbology, College of Korean Medicine, Kyung Hee University. Specimens were deposited in the herbarium of the anatomy laboratory, College of Korean Medicine, Kyung Hee University. The extract was prepared by decocting 300 g of the dried herb with 3 L of boiling distilled water for 2 h and filtered using Whatman No. 3 filter paper. The filtrate was concentrated by evaporation under reduced pressure and lyophilized, yielding 27.3 g dried powder (yield ratio 9.1%). The extract was stored at -20°C until use, and dissolved in water just before use.

Quantitative evaluation of BF extract was performed using a reference compound, Saikosaponin A (≥95%, Sigma-Aldrich, St. Louis, MO, United States), by high-performance liquid chromatography (HPLC) equipped with a dual λ absorbance detector (Waters 2487) on the Waters 2695 system (Waters, Milford, MA, United States). Four hundred milligrams of BF extract was dissolved in 10 mL of HPLC-grade methanol by sonication for 30 s. This was filtered using a 0.2 μm Membrane filter (Millipore, Watford, United Kingdom), and 10 μL was injected into the HPLC system. The separation was performed on a C-18 Symmetry column (5 μm, 4.6 × 150 mm, Waters). The mobile phase was acetonitrile (A) and water (B) at a constant composition of 40% A from 0 to 25 min. The flow rate was 1.0 mL/min, and the detector was set at 215 nm at 30°C. The peak of saikosaponin A in the BF extract was synchronized with the standard (Supplementary Figure S1). The concentration of saikosaponin A in the BF extract was 6.66 μg/mg (0.67%).

### Animals

Female Sprague-Dawley rats (age 11 weeks; body weight 240–250 g), obtained from Nara Biotech (Seoul, South Korea), were maintained at 21–23°C and 45–65% relative humidity with a 12 h light-dark cycle and free access to food and water. Animal maintenance and treatment were carried out in accordance with the relevant guidelines and regulations issued by Kyung Hee University. All of the animal experimental procedures were approved by the Kyung Hee University Institutional Animal Care and Use Committee (KHUASP(SE)-15-101).

### Surgical Procedures and Experimental Design

Following a 1 week acclimatization, the rats were randomly assigned to two groups, with one group undergoing ovariectomy (OVX, *n* = 32), and the other group subjected to sham surgery (SHAM, *n* = 8). OVX was performed on 12 weeks old rats under isoflurane anesthesia. Both ovaries were excised through a small abdominal incision. The sham-operated rats underwent the same procedure, except the ovaries were not removed. After surgery, gentamicin (10 mg/kg) was administered once a day for 3 days to prevent infection. A day after surgery, the OVX rats were divided into the following groups (*n* = 8 per group): one with no further treatment (OVX control group), two groups administered BF at doses of either 12 or 120 mg/kg body weight (BF-L and BF-H, respectively) and one that received 17β-estradiol at a dose of 100 μg/kg body weight (E2 group), serving as a positive control. BF extract and E2 were dissolved in 0.9% saline and administered orally once a day for 8 weeks. SHAM and OVX rats were treated with an equivalent volume of 0.9% saline as a vehicle. The final administration was performed 2 h before euthanasia.

Body weight was measured weekly throughout the experiment. At the end of the experiment, the uterus was dissected and immediately weighed. Uterine index (mg/g body weight) was calculated by dividing the uterine weight by the body weight. Both femurs and tibias were also dissected, immediately weighed, and stored appropriately for further measurement. To reduce the individual body weight differences, the relative weights (% of body weight) were calculated at sacrifice.

### Measurements of Serum Biochemical Parameters

On the last day of treatment administration, blood from pentobarbital-anesthetized rats was collected through cardiac puncture, clotted at room temperature for 30 min, then centrifuged for 30 min at 1,000 × *g* at 4°C to remove the clot. Serum osteocalcin was measured with a commercial enzyme immunoassay kit (Biomedical Technology, Stoughton, MA, United States) according to the manufacturer’s protocol. Serum TRAP activity was quantitated by colorimetric analysis (details are described below).

### Physical Properties of Bone

The physical properties of bone were determined using bone weight and density, which reflect mineral deposition and mechanical strength. After measuring the wet weight of tibial bone using a scale, the bone was ashed in a muffle furnace at 900°C for 18 h. Ashed samples were weighed after cooling completely in a desiccator. The bone density of the right femurs was measured by the method based on Archimedes’ principle, as described previously (Kim et al., 2016). Briefly, right femurs were placed in vials filled with deionized water. The vials were placed in a vacuum for 90 min to ensure that all trapped air diffused from the bones. They were removed from the vials, dried with gauze, weighed, and placed in new vials containing deionized water. The bones were reweighed in the water. Bone density was calculated as femur mass divided by volume (g/cm^3^).

### Histological Analysis of Trabecular Bone

Histological analysis of trabecular bone was performed to evaluate changes in bone tissue. The left proximal femurs were fixed in 10% neutral buffered formalin for 48 h, decalcified in 10% ethylenediaminetetraacetic acid (EDTA) for 3 weeks, and embedded in paraffin. Five-micrometer-thick longitudinal sections were stained with hematoxylin and eosin (H&E), followed by measurement of bone total surface area (T.Ar), trabecular bone area (Tb.Ar), and trabecular pemimeter (Th.Pm) with ImageJ software (NIH, Bethesda, MD, United States) in the proximal femoral metaphysis in a region that is 900 μm wide × 660 μm high, 1 mm below the growth plate of each section. The bone volume fraction (BV/TV; the percent of trabecular area relative to the total area), trabecular thickness (Tb.Th), trabecular number (Tb.N), and trabecular space (Tb.Sp) were calculated according to the Parfitt’s formulas (Dempster et al., 2013; Lei et al., 2017); BV/TV =Tb, Ar/T.Ar × 100; Tb.Th = (2000/1.199) × (Th.Ar/Tb.Pm); Tb-N = (1.199/2) × (Tb.Pm/T.Ar); Tb-Sp = (2000/1.199) × (T.Ar-Tb.Ar)/Tb.Pm.

### Immunohistochemistry

Immunohistochemical analysis was performed using the standard manufacturer recommended protocol. Briefly, 5 μm-thick longitudinal sections of the proximal femurs were antigen-retrieved by enzymatic digestion with 0.4 mg/mL proteinase K for 8 min at 37°C, and then stained with antibody against iNOS (diluted 1:50) overnight at 4°C. An avidin-biotin immunoperoxidase system was subsequently used to detect immunoactivity according to the manufacturer’s instructions (Vectastain Elite ABC kit; Vector Laboratories, Burlingame, CA, United States) followed by counterstaining with methyl green. The sections were examined under a light microscope (original magnification, 100×).

### Cell Culture

Murine RAW 264.7 macrophage cells, obtained from the Korean Cell Line Bank (Seoul, South Korea), were cultured in Dulbecco’s Modified Eagle’s Medium (DMEM; Life Technologies, Carlsbad, CA, United States) containing 10% fetal bovine serum (FBS; Life Technologies) and 100 units/mL penicillin/streptomycin (Life Technologies) at 37°C in a humidified incubator with 5% CO_2_.

### Differentiation of RAW 264.7 Cells Into Osteoclasts

RAW 264.7 cells were differentiated into OCs with 100 ng/mL RANKL (Peprotech, Rocky Hill, NJ, United States) in α-MEM containing 10% FBS for 5-7 days, with replacement of fresh medium every 2 days. OCs were detected by TRAP staining. TRAP-positive multinucleated (>3 nuclei) cells were identified as mature OCs.

### TRAP Assay for Osteoclasts

OCs were identified by quantitating TRAP activity using either cytochemical staining or a colorimetric analysis. For cytochemical staining of TRAP-positive cells, deparaffinized tissue sections or 10% formaldehyde-fixed cells were stained for TRAP using a commercial acid phosphatase leukocyte kit (Sigma-Aldrich, St. Louis, MO, United States) following the manufacturer’s protocol. TRAP-positive cells with > 3 nuclei were counted as OCs under an inverted microscope (Olympus, Tokyo, Japan). Colorimetric analysis for TRAP activity in serum and culture supernatants was based on the ability of phosphatases to catalyze the hydrolysis of the *p*-nitrophenyl phosphate (pNPP) substrate to *p*-nitrophenol, a chromogenic product with absorbance at 405 nm. Briefly, samples (50 μL) were incubated with an equal volume of substrate (12.5 mM pNPP in 50 mM sodium tartrate and 0.12 M sodium acetate, pH 5.2) for 60 min at 37°C. The reaction was stopped with 0.9 M NaOH and the absorbance was measured at 405 nm with a microplate reader. Results are expressed as optical density (OD) values.

### Cell Viability Assay

Cytotoxicity was determined using a water-soluble tetrazolium salt (WST-1)-based EZ-Cytox cell viability assay kit (Daeil Lab Service Co., Seoul, South Korea) according to the manufacturer’s protocol. Absorbance was read at 450 nm using a microplate reader (Molecular Devices, Sunnyvale, CA, United States). Results are expressed as percentages of the untreated control.

### *In vitro* Bone Resorption Assays

Bone resorption activity of OCs was assessed by pit formation assay using the Corning Osteo Assay Surface (Corning, Inc., Corning, NY, United States). Briefly, RAW 264.7 cells were plated in 96-well Corning Osteo Assay Surface Microplates at 5 × 10^3^ cells/well and treated with BF in the presence of RANKL (100 ng/mL) for 7 days. The medium containing RANKL and BF was replaced every 2nd day. Subsequently, the cells were removed completely by washing with 10% bleach solution. Resorption pits were observed under a light microscope (original magnification, 100×) and the resorption area was measured using ImageJ software (NIH). The results are expressed as a percentage of the total plate area.

### MMP Activity

MMP-9 activity in cell culture supernatants was measured by gelatin zymography. Culture supernatants were subjected to electrophoresis on a 10% polyacrylamide gel containing 1% gelatin. After electrophoresis, gels were incubated for 24 h at 37°C in 50 mM Tris-HCl (pH 7.5) containing 5 mM CaCl_2_ and 0.02% NaN_3_, and stained with Coomassie Brilliant Blue G-250 (Sigma-Aldrich, St. Louis, MO, United States). Unstained bands corresponding to gelatinolytic activity were quantitated using ImageJ software (NIH).

### Semi-quantitative Reverse Transcriptase Polymerase Chain Reaction (RT-PCR)

Total RNA was extracted using the TakaRa RNAiso Plus reagent (TaKaRa, Tokyo, Japan) according to the manufacturer’s instructions. Complementary DNA (cDNA) was made from 2 μg of total RNA using the SuperScript III First-Strand Synthesis System kit (Invitrogen, Carlsbad, CA, United States) and random hexamers. cDNA samples were then amplified using Taq Polymerase (TaKaRa). Primer sequences and PCR conditions are given in Supplementary Table S1. PCR products were subjected to electrophoresis on 1% agarose gel and stained with ethidium bromide. Band intensities were quantified with ImageJ software (NIH) and normalized to values for GAPDH. Results are expressed as fold induction relative to those of cells treated with RANKL alone.

### Western Blot Analysis

Total proteins were extracted with RIPA lysis buffer containing mammalian protease inhibitor cocktail and phosphatase inhibitor cocktails 2 and 3 (Sigma-Aldrich, St. Louis, MO, United States). Nuclear and cytoplasmic fractions were prepared using the NE-PER Nuclear and Cytoplasmic Extraction Reagents kit (Thermo Fisher Scientific, Waltham, MA, United States) according to the manufacturer’s protocol. Protein concentration was determined using the Bradford Assay (BioRad, Hercules, CA, United States). Equal amounts of protein were subjected to sodium dodecyl sulfate polyacrylamide gel electrophoresis (SDS-PAGE) followed by transfer to nitrocellulose membranes. After blocking with 5% dried milk, the proteins were probed with primary antibodies specific for iNOS (1:200, Santa Cruz Biotechnology, Santa Cruz, CA, United States), inhibitor of kappa B α (IκBα; 1:1000, Cell Signaling, Beverly, MA, United States), phospho-IκBα (1:1000, Cell Signaling, Beverly, MA, United States), NF-κB p65 (1:1000, Cell Signaling, Beverly, MA, United States), phospho- or total MAPK (1:1000, Cell Signaling, Beverly, MA, United States), and HO-1 (1:5000, GeneTex, Irvine, CA, United States), and then with the appropriate HRP-conjugated secondary antibody. Immunoreactive bands were detected using enhanced chemiluminescence (ECL) reagents (Thermo Fisher Scientific, Waltham, MA, United States) and photographed. To verify equal loading, blots were stripped in stripping buffer and re-probed with antibodies against β-actin (1:500, Santa Cruz Biotechnology, Santa Cruz, CA, United States) or lamin B (1:500, Santa Cruz Biotechnology, Santa Cruz, CA, United States). Band intensities were measured using ImageJ software (NIH).

### Statistical Analysis

Data are presented as means ± standard error of the mean (SEM). All statistical analyses were performed using IBM SPSS version 23 (IBM Corp., Armonk, New York, United States). Two-tailed unpaired *t*-tests were used to compare changes in the levels of target genes or proteins at each time point. One-way analysis of variance (ANOVA) followed by Tukey’s *post hoc* test was used to identify significant differences among three or more groups. To evaluate for differences across time, the results of body weight were analyzed by one-way repeated-measures ANOVA followed by Tukey’s *post hoc* test. Differences with *P* < 0.05 were considered significant, while *P* < 0.1 was considered a trend toward significance.

## Results

### Oral Administration of BF Extract Ameliorated OVX-Induced Body Weight Gain, but Not Uterine Atrophy

**FIGURE 1:**
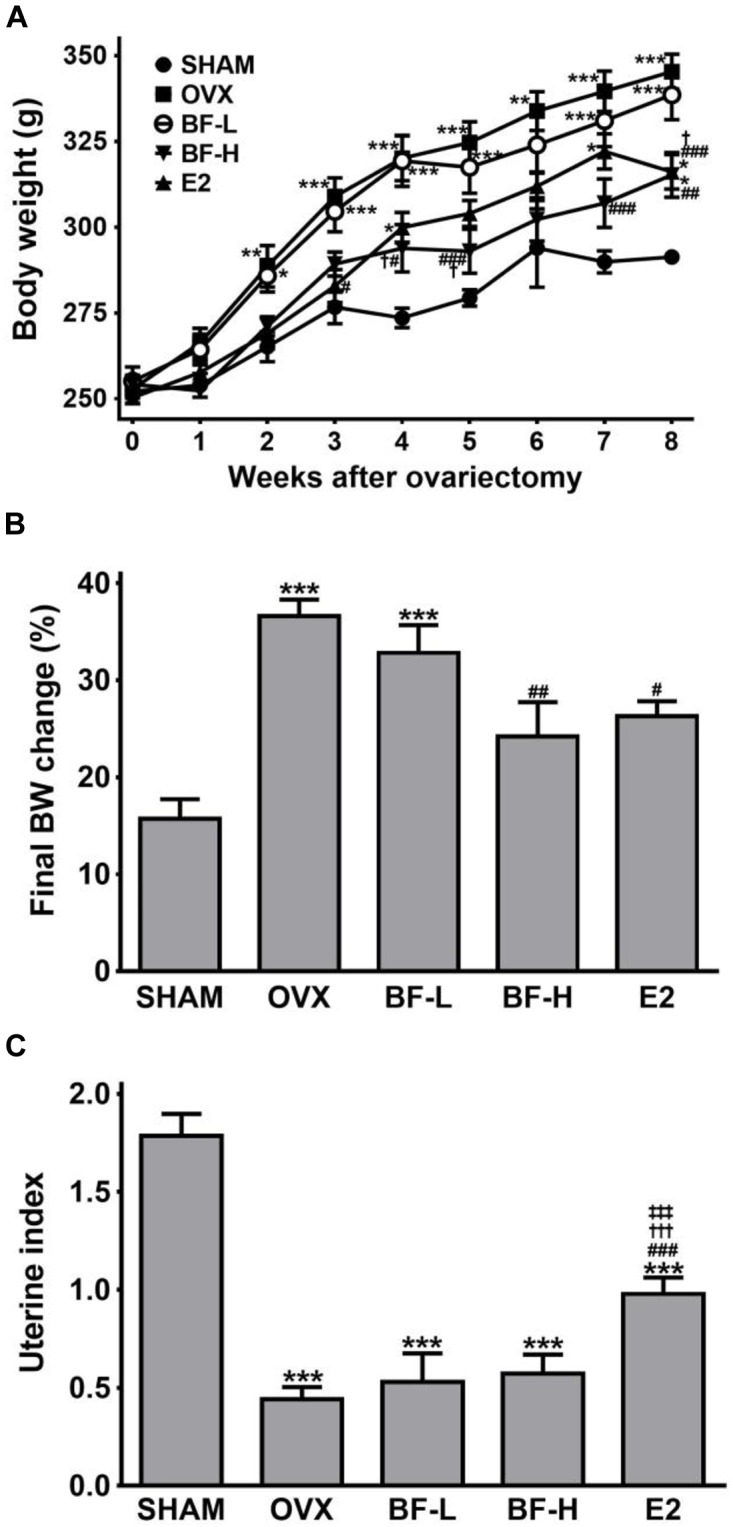
Effects of BF extract on body weight and uterine index in ovariectomized rats. **(A)** Body weight was measured weekly for 8 weeks following ovariectomy. **(B)** The final body weight gain was calculated as the percentage of weight gain relative to initial body weight. **(C)** Uterine atrophy was assessed by the uterine index, represented as uterine weight divided by body weight. SHAM, sham-operated control; OVX, untreated ovariectomized rats; BF-L, ovariectomized rats treated with low-dose BF (12 mg/kg); BF-H, ovariectomized rats treated with high-dose BF (120 mg/kg); E2, ovariectomized rats treated with 17β-estradiol (100 μg/kg). Values are expressed as means ± standard error of the mean (SEM). ^∗^*P* < 0.05, ^∗∗^*P* < 0.01, ^∗∗∗^*P* < 0.001 versus SHAM group; ^#^*P* < 0.05, ^##^*P* < 0.01, ^###^*P* < 0.001 versus OVX group; ^†^*P* < 0.05, ^†††^*P* < 0.001 versus BF-L group; ^‡‡‡^*P* < 0.001 versus BF-H group.

OVX induces a marked body weight gain with uterine atrophy. Therefore, to confirm successful OVX and to investigate whether BF ameliorates OVX-induced changes in body weight and uterine size, we initially assessed body weight and uterine weight. The initial body weights did not differ among the groups. The body weights gradually increased over time in all groups, but OVX rats gained significantly more weight than the SHAM animals. The BF-H group, but not BF-L, weighed significantly less than the OVX group throughout the study (Figure 1A). Exogenous E2 treatment tended to prevent body weight gain after OVX during the study period, but it was not significant except at Week 3 (Figure 1A). At the end of the study, only the body weight gain of the BF-H group was significantly less than the OVX control group (Figure 1B). As expected, OVX resulted in reduced uterine weight relative to SHAM controls, indicating successful OVX. E2 treatment was markedly effective in preventing uterine atrophy by OVX, whereas BF administration had no effect on uterine weight (Figure 1C).

### Oral Administration of BF Extract Ameliorated Bone Loss in OVX Rats

**FIGURE 2:**
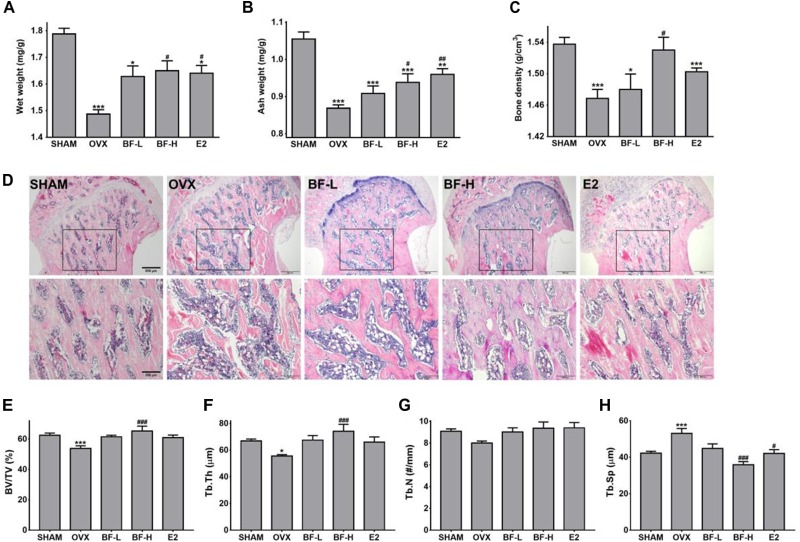
Effects of BF extract on bone loss in ovariectomized rats. **(A)** Tibias were weighed before ashing. **(B)** The ash weight of tibias was measured to determine mineral content. Tibia weight before and after ashing is presented relative to body weight. **(C)** Bone density was determined in femurs using Archimedes’ principle. **(D)** Longitudinal sections of proximal femoral metaphysis were stained with hematoxylin and eosin (H&E). Lower panels are enlarged images of boxed areas in upper panels. Scale bar in upper panels = 500 μm, in lower panels = 200 μm. **(E–H)** The extent of bone resorption was assessed by calculating the percent of trabecular area relative to the total area (BV/TV), trabecular thickness (Tb.Th), trabecular separation (Tb.Sp), and number of trabeculae (Tb.N) in proximal femoral metaphysis. SHAM, sham-operated control; OVX, untreated ovariectomized rats; BF-L, ovariectomized rats treated with low-dose BF (12 mg/kg); BF-H, ovariectomized rats treated with high-dose BF (120 mg/kg); E2, ovariectomized rats treated with 17β-estradiol (100 μg/kg). Values are expressed as means ± SEM. ^∗^*P* < 0.05, ^∗∗^*P* < 0.01, ^∗∗∗^*P* < 0.001 versus SHAM group; ^#^*P* < 0.05, ^##^*P* < 0.01, ^###^*P* < 0.001 versus OVX group.

We next investigated whether oral administration of BF extract protects OVX rats against osteoporotic symptoms characterized by bone loss, as measured by bone weight and density. Compared with the SHAM rats, OVX rats exhibited significantly decreased tibial wet weight. However, daily dosing with BF extract or E2 for 8 weeks elicited significant increases in bone wet weight compared with OVX control rats (Figure 2A). Although similar trends were found in the change in the mineralized bone mass of tibias measured by ashing, only high dose (120 mg/kg) BF- or E2-treated rats had significantly higher tibial ash contents (Figure 2B). We also assessed bone density, an important indicator of bone quality and strength. The decrease in femoral bone density caused by OVX was unaffected by the administration of BF or E2, but a significant difference was detected only in the BF-H group (Figure 2C). The deterioration of trabecular architecture is a contributory factor to bone fragility, and its disruption accelerates after menopause. Therefore, to further confirm the effects of BF on OVX-induced bone loss, trabecular microstructure was next assessed by histological examination of proximal femurs. As shown in Figure 2D, microscopic examination of metaphyseal trabecular bone in the proximal femurs of SHAM-operated rats revealed normal, dense, and uniform trabecular bone architecture, whereas those of OVX-control rats exhibited disruptive changes, sparse or irregular arrangement, or incomplete formation of a connective network. Daily dosing with BF for 8 weeks greatly improved OVX-induced trabecular bone quality, with much wider and more complete trabeculae, especially in the BF-H group. E2 also significantly protected against trabecular bone loss induced by OVX, similar to the effects observed in the BF-L group. For quantitative analysis of morphological changes in the trabecular structure, bone microarchitecture parameters, including BV/TV (the percent of trabecular area relative to the total area), Tb.Th (trabecular thickness), Tb.N (trabecular number), and Tb.Sp (trabecular separation), were measured. Compared to the OVX group, BV/TV (Figure 2E), and Tb.Th (Figure 2F) in high dose of BF-treated OVX rats were significantly increased, while there was a statistically significant decrease in Tb.Sp level (Figure 2H). Tb.N was unchanged with BF treatment (Figure 2G). Compared to the OVX group, E2 also significantly decreased Tb.Sp levels (Figure 2H). Taken together, these data indicate that BF may alleviate bone loss induced by OVX.

### Oral Administration of BF Extract Attenuated Osteoclast Activity in OVX Rats

**FIGURE 3:**
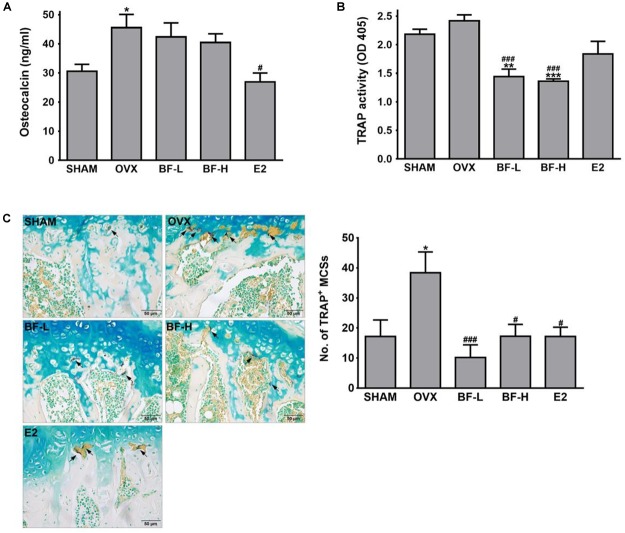
Effects of BF extract on bone formation and resorption in ovariectomized rats. **(A)** Bone formation was assessed by measuring serum osteocalcin by enzyme-linked immunosorbent assay (ELISA). **(B)** Serum tartrate-resistant acid phosphatase (TRAP) activity as a bone resorption marker was measured by colorimetric analysis using the *p*-nitrophenyl phosphate (pNPP) substrate. **(C)** Proximal femurs were assessed by TRAP staining for the identification of osteoclasts. Arrows, TRAP-positive multinucleated cells (TRAP^+^MNCs; ≥ 3 nuclei). Scale bar = 50 μm. TRAP^+^MNCs in femoral epiphysis were counted as mature osteoclasts. SHAM, sham-operated control; OVX, untreated ovariectomized rats; BF-L, ovariectomized rats treated with low-dose BF (12 mg/kg); BF-H, ovariectomized rats treated with high-dose BF (120 mg/kg); E2, ovariectomized rats treated with 17β-estradiol (100 μg/kg). Values are expressed as means ± SEM. ^∗^*P* < 0.05, ^∗∗^*P* < 0.01, ^∗∗∗^*P* < 0.001 versus SHAM group; ^#^*P* < 0.05, ^###^*P* < 0.001 versus OVX group.

Bone loss occurs due to an imbalance of osteoblastic bone formation and osteoclastic bone resorption; typically,an excess of resorption over formation. To address whether the anti-osteoporotic activity of BF is mediated through the promotion of osteoblastic bone formation and/or suppression of excessive osteoclastic bone resorption, we next measured the serum osteocalcin concentration as an osteoblast-specific bone turnover marker (Singh et al., 2015), and serum TRAP activity as an OC-specific bone resorption marker. The OVX group showed a significantly higher serum osteocalcin level than the SHAM group. On the other hand, contrary to expectations, serum TRAP activityin the OVX was slightly but not significantly increased compared to those of the SHAM group, which seems to be due to the possible lack of sensitivity of the assay used. Unlike E2, which significantly decreased both osteocalcin levels and TRAP activity in serum compared with levels in the OVX control, treatment with BF at both low and high doses significantly decreased TRAP activity, but did not affect serum osteocalcin levels (Figures 3A,B). This suppressive effect of BF on bone resorption was further confirmed by the decrease in the numbers of OCs in the proximal femoral metaphysis compared with those in OVX rats, as detected by histochemical staining for TRAP (Figure 3C) and by immunohistochemical staining for cathepsin K, one of the genes typically expressed by OCs (Supplementary Figure S2). These results indicate that BF may affect bone resorption rather than bone formation.

### BF Extract Inhibited RANKL-Induced Osteoclast Formation *in vitro*

**FIGURE 4:**
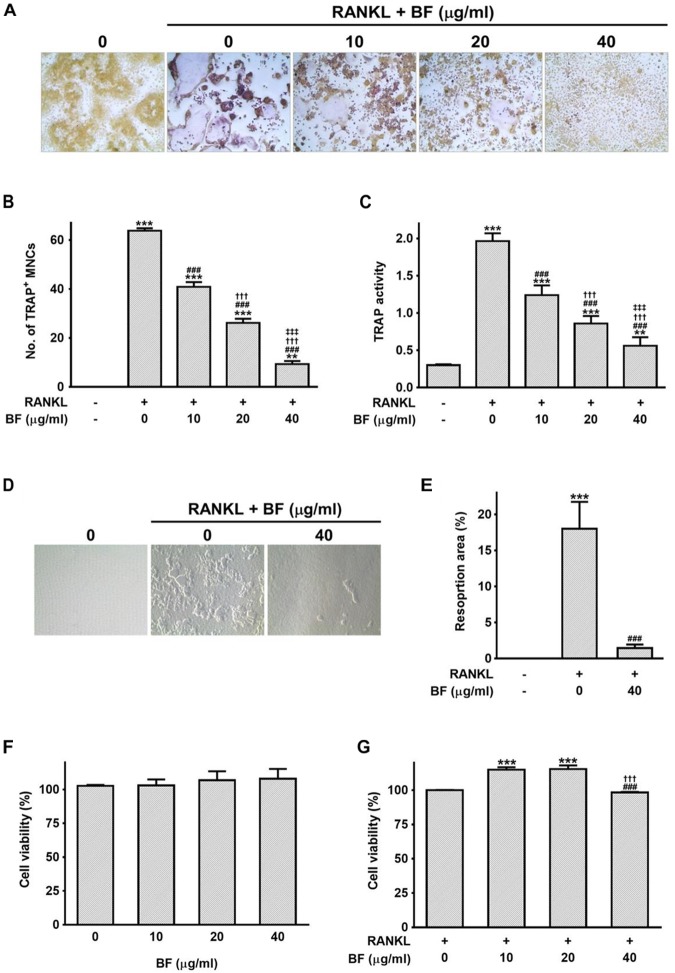
Effects of BF extract on osteoclast formation and subsequent activity in RANKL-stimulated RAW 264.7 cells. **(A)** RANKL-induced osteoclast differentiation of RAW 264.7 cells into osteoclasts was assessed by TRAP staining 5 days after treatment with RANKL (100 ng/mL) in the presence or absence of BF extract and **(B)** TRAP-positive multinucleated cells (TRAP+MNCs; ≥ 3 nuclei) were counted as mature osteoclasts. Original magnification, 100×. **(C)** TRAP activity was determined in the culture medium using pNPP as the substrate. **(D)** Osteoclasts activity was assessedby pit formation assay. RAW 264.7 cells were cultured on Osteo Assay Surfaces with RANKL (100 ng/mL) for 7 days in the presence or absence of BF extract. The resorption pits were visualized by light microscopy. Original magnification, 100×. **(E)** The resorption area was measured and expressed as a percentage of the totalarea analyzed. **(F,G)** The cytotoxicity of BF extract in RAW 264.7 cells was evaluated by water-soluble tetrazolium salt (WST) assay. Cells were cultured with BF extract alone for 24 h **(F)** or BF in the presence of RANKL (100 ng/mL) for 72 h **(G)**. Cell viability is expressed as a percentage of the untreated control. All data are expressed as means ± SEM. Experiments were performed with at least three independent replicates. ^∗∗^*P* < 0.01, ^∗∗∗^*P* < 0.001 versus untreated control; ^###^*P* < 0.001 versus RANKL alone; ^†††^*P* < 0.001 versus RANKL+BF (10 μg/mL); ^‡‡‡^*P* < 0.001 versus RANKL+BF (20 μg/mL).

To address the direct effects of BF on OC formation, its effects on RANKL-induced OC formation in RAW 264.7 cells were investigated by TRAP staining and a pit formation assay. RANKL treatment induced the differentiation of RAW 264.7 cells into OCs, represented by multinucleated TRAP^+^ cells. In contrast, when the cells were treated with both RANKL and BF, the number of TRAP-positive multinucleated OCs and TRAP activity were significantly suppressed in a dose-dependent manner, compared to the cells treated with RANKL alone (Figures 4A–C). In addition, RANKL-induced OCs yielded a moderate resorption pit area, whereas BF-treated cells exhibited markedly decreased resorption activity (Figure 4D). The resorption area of 40 μg/mL BF-treated OCs was significantly lower than that of untreated OCs (Figure 4E). These results indicated that BF inhibits the RANKL-induced OC formation. None of the concentrations of BF tested affected the viability of RAW 264.7 cells (Figures 4F,G), excluding the possibility that its osteoclastogenesis inhibition was due to cytotoxicity.

### BF Extract Reduced the Expression of Genes Critical for Osteoclast Formation and Function

**FIGURE 5:**
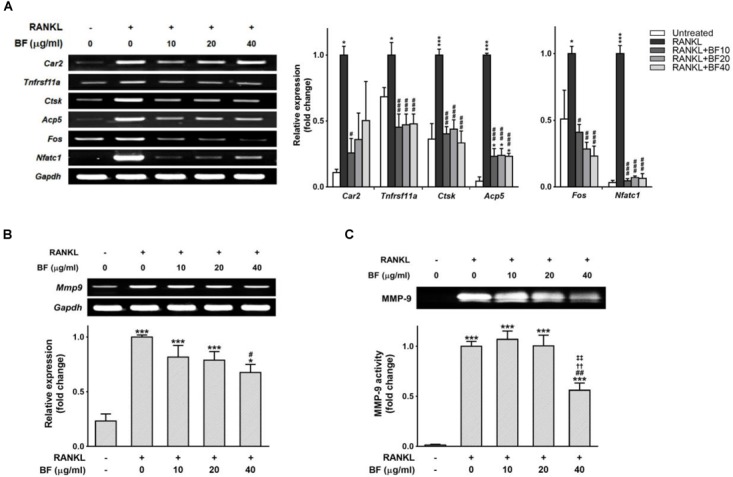
Effects of BF extract on osteoclast-specific functional genes in RANKL-stimulated RAW 264.7 cells. RAW 264.7 cells were treated with RANKL in the presence or absence of BF for 4 days. **(A)** Osteoclasts derived from RANKL-stimulated RAW 264.7 cells were assayed for expression of the indicated genes by semi-quantitative RT-PCR (left). The mRNA expression levels of osteoclast-specific genes (middle) and RANKL-induced transcription factors (right) were normalized to those of *Gapdh* mRNA and are presented relative to those in RANKL-treated cells. *Car2*, carbonic anhydrase 2; *Tnfrsf11a*, RANK; *Ctsk*, cathepsin K; *Acp5*, TRAP; *Fos*, c-fos osteosarcoma oncogene; *Nfatc1*, nuclear factor of activated T-cells, cytoplasmic, calcineurin-dependent 1; *Gapdh*, glyceraldehyde 3-phosphate dehydrogenase. **(B)** RAW 264.7 cells treated with RANKL in the presence or absence of BF for 4 days were assessed for *Mmp9* expression by semi-quantitative RT-PCR (top). mRNA abundance of *Mmp9* was normalized to that of *Gapdh* mRNA and presented relative to that in RANKL-treated cells (bottom). *Mmp9*, matrix metalloproteinase-9; *Gapdh*, glyceraldehyde 3-phosphate dehydrogenase. **(C)** RAW 264.7 cells were treated with RANKL in the presence or absence of BF for 5 days, and the culture supernatants were analyzed for MMP-9 proteolytic activity by gelatin zymography (top). The proteolytic activity of MMP-9 is shown relative to that in RANKL-treated cells (bottom). All data are expressed as means ± SEM. Experiments were performed with at least three independent replicates. ^∗^*P* < 0.05, ^∗∗∗^*P* < 0.001 versus untreated control; ^#^*P* < 0.05, ^##^*P* < 0.01, ^###^*P* < 0.001 versus RANKL alone; ^††^*P* < 0.01 versus RANKL+BF (10 μg/mL); ^‡‡^*P* < 0.01 versus RANKL+BF (20 μg/mL).

Mature OCs are also characterized by the expression of OC-specific gene markers that are crucial for bone organic matrix degradation and bone resorption, such as RANK, carbonic anhydrase 2, cathepsin K, TRAP, and MMP-9 (Teitelbaum and Ross, 2003). The expression levels of these genes greatly increased in RANKL-treated RAW 264.7 cells, but co-treatment with BF markedly blocked RANKL-induced upregulation of these genes (Figure 5A). Subsequently, we also tested the effects of BF on the mRNA expression and activity of MMP-9, one of the most potent proteolytic effectors of OC-mediated bone resorption (Sundaram et al., 2007), in RANKL-stimulated RAW 264.7 cells. Consistent with the results of the TRAP assay and pit formation assay, the mRNA expression of MMP-9 was suppressed in a concentration-dependent manner by BF treatment (Figure 5B). In addition, we also confirmed by gelatin zymography that BF treatment suppressed the activity of MMP-9 (Figure 5C). Collectively, these results suggest that BF suppresses OC differentiation and function at the transcriptional level.

### BF Negatively Regulated RANKL-Induced Expression of c-Fos and NFATc1

**FIGURE 6:**
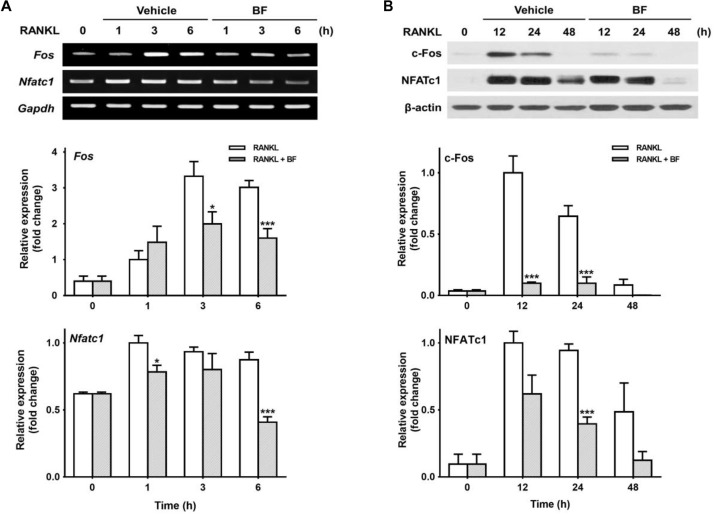
Effects of BF extract on the expression of osteoclast-specific transcription factors in RANKL-stimulated RAW 264.7 cells. RAW 264.7 cells were treated with RANKL (100 ng/mL) in the presence or absence of BF (40 μg/mL) for the indicated time periods. **(A)** *Fos* and *Nfatc1* mRNA levels were assessed by semi-quantitative RT-PCR (top), normalized to those of *Gapdh* mRNA, and expressed as fold induction relative to those of cells treated with RANKL alone (middle and bottom, respectively). *Fos*, c-fos osteosarcoma oncogene; *Nfatc1*, nuclear factor of activated T-cells, cytoplasmic, calcineurin-dependent 1; *Gapdh*, glyceraldehyde 3-phosphate dehydrogenase. **(B)** Protein expression levels of c-Fos and NFATc1 were evaluated by western blot analysis (top), normalized to those of β-actin, and expressed as fold induction relative to that in cells treated with RANKL alone (middle and bottom, respectively). All data are expressed as means ± SEM. Experiments were performed with at least three independent replicates. ^∗^*P* < 0.05, ^∗∗∗^*P* < 0.001 versus time-matched RANKL-treated controls.

Given that c-Fos and NFATc1 are two major transcription factors that play a critical role in the regulation of OC differentiation genes (Grigoriadis et al., 1994; Takayanagi, 2007b), we tested the effects of BF on RANKL-induced c-Fos and NFATc1 expression to examine whether BF inhibits RANKL-induced OC differentiation by downregulating c-Fos and NFATc1. As shown in Figure 5A, the abundance of c-Fos and NFATc1 mRNA increased in response to RANKL, but BF strongly inhibited this increased expression of *c-Fos* and *Nfatc1*, even at the low concentration of 10 μg/mL, in RANKL-stimulated RAW 264.7 cells. Similar results were also obtained in a time course experiment. The abundance of c-Fos and NFATc1 mRNA was upregulated when RAW 264.7 cells were stimulated with RANKL for the indicated time points, but significantly reduced by co-treatment with BF (Figure 6A). Similarly, BF suppressed c-Fos and NFATc1 protein expression (Figure 6B). These results suggest that the inhibitory effects of BF on OC differentiation are mediated, at least in part, by the downregulation of c-Fos and NFATc1 in RAW 264.7 cells.

### BF Positively Regulated RANKL-Induced Expression of IFN-β and iNOS/NO

**FIGURE 7:**
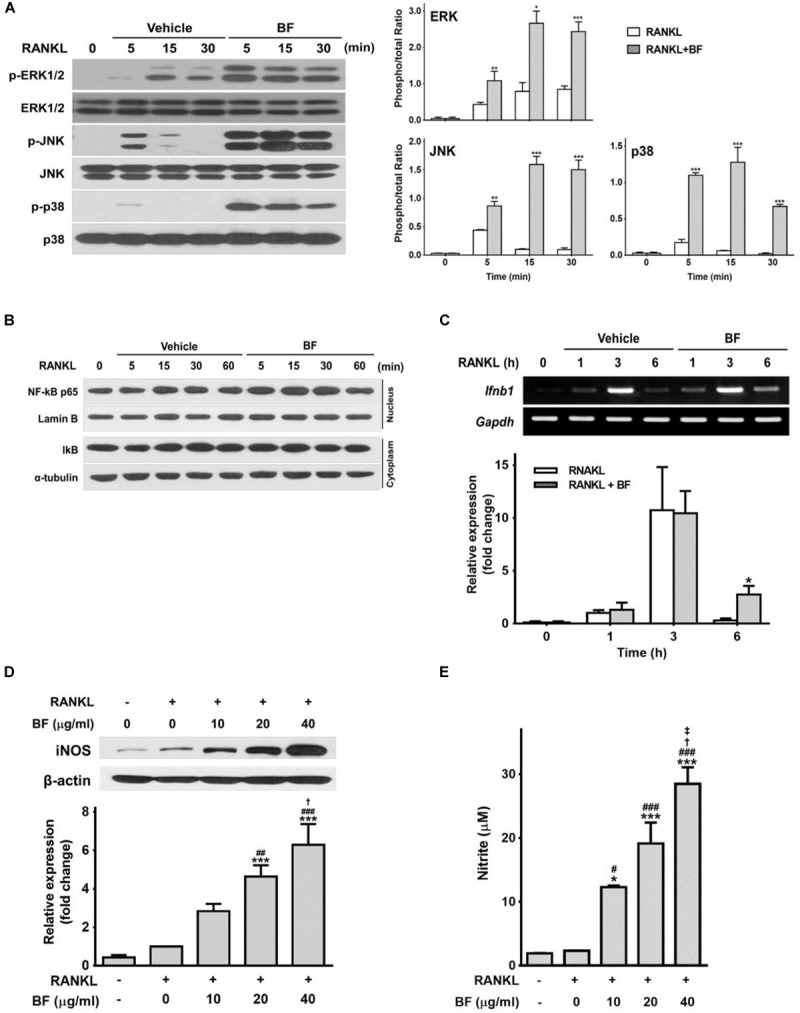
Effects of BF extract on the expression of IFN-β and iNOS and the release of nitric oxide in RANKL-stimulated RAW 264.7 cells. **(A–C)** RAW 264.7 cells were treated with RANKL (100 ng/mL) in the presence or absence of BF (40 μg/mL) for the indicated time periods. **(A)** Total cell protein extracts were analyzed by western blot for p-ERK 1/2, ERK 1/2, p-JNK, JNK, p-p38, and p38, and normalized to those of total form. Blots are representative of 3 independent experiments. **(B)** NF-κB subunit p65 and Lamin B protein levels in nuclear extracts was determined by western blot. IκBα and α-tubulin levels were detected in cytosolic extracts. Blots are representative of 3 independent experiments. **(C)** *Ifnb1* mRNA levels were assessed by semi-quantitative RT-PCR (top), normalized to those of *Gapdh* mRNA, and expressed as fold induction relative to those in cells treated with RANKL alone (bottom). *Ifnb1*, interferon beta 1; *Gapdh*, glyceraldehyde 3-phosphate dehydrogenase. **(D)** RAW 264.7 cells were treated with RANKL (100 ng/mL) in the presence or absence of BF for 3 days. Protein expression of inducible nitric oxide synthase (iNOS) was evaluated by western blot analysis (top), normalized to that of β-actin, and expressed as fold induction relative to that in cells treated with RANKL alone (bottom). **(E)** Nitric oxide production was determined as the accumulation of nitrite, estimated by Griess assay, in supernatants from cells after treatment for 3 days with RANKL (100 ng/mL) in the presence or absence of BF. All data are expressed as means ± SEM. Experiments were performed with at least three independent replicates. ^∗^*P* < 0.05, ^∗∗^*P* < 0.01, ^∗∗∗^*P* < 0.001 versus untreated control; ^#^*P* < 0.05, ^##^*P* < 0.01, ^###^*P* < 0.001 versus RANKL alone; ^†^*P* < 0.05 versus RANKL+BF (10 μg/mL); ^‡^*P* < 0.05 versus RANKL+BF (20 μg/mL).

MAPKs, particularly p38, and NF-κB, contribute to the regulation of c-Fos and NFATc1 upon RANKL stimulation (Matsumoto et al., 2000; Boyle et al., 2003; Lee et al., 2016). To determine whether MAPKs or NF-κB are involved in the inhibitory effects of BF on osteoclastogenesis as an earlier signaling event, we next tested the effects of BF on RANKL-induced activation of MAPKs and NF-κB. Contrary to our expectations, BF did not inhibit but rather markedly augmented RANKL-induced MAPK phosphorylation (Figure 7A). Furthermore, it did not affect RANKL-induced degradation of IκBα in the cytosol and nuclear translocation of the NF-κB p65 subunit (Figure 7B).

We next determined, using semi-quantitative RT-PCR, whether the inhibitory effect of BF is associated with the expression of IFN-β, another major factor regulating bone homeostasis through an autocrine negative feedback loop via c-Fos inhibition (Takayanagi et al., 2002). The abundance of IFN-β mRNA increased after RANKL treatment, peaking at 3 h and decreasing thereafter. Although a similar pattern was observed in RAW 264.7 cells co-treated with RANKL and BF, the abundance of IFN-β mRNA after co-treatment with RANKL and BF for 6 h remained significantly higher than that in cells treated with RANKL alone (Figure 7C). As RANKL-induced IFN-β triggers iNOS-derived NO production as important signals that may negatively influence the extent of OC formation and differentiation into bone pit-resorbing cells (Zheng et al., 2006), additional experiments were carried out to examine the effects of BF on iNOS expression and NO release in RANKL-treated RAW 264.7 cells. Treatment with RANKL in the presence of BF significantly upregulated the expression of iNOS protein and the production of NO, in a dose-dependent manner (Figures 7D,E). These results raise the possibility that BF might negatively regulate osteoclastogenesis through the upregulation of IFN-β and subsequent iNOS-derived NO production.

**FIGURE 8:**
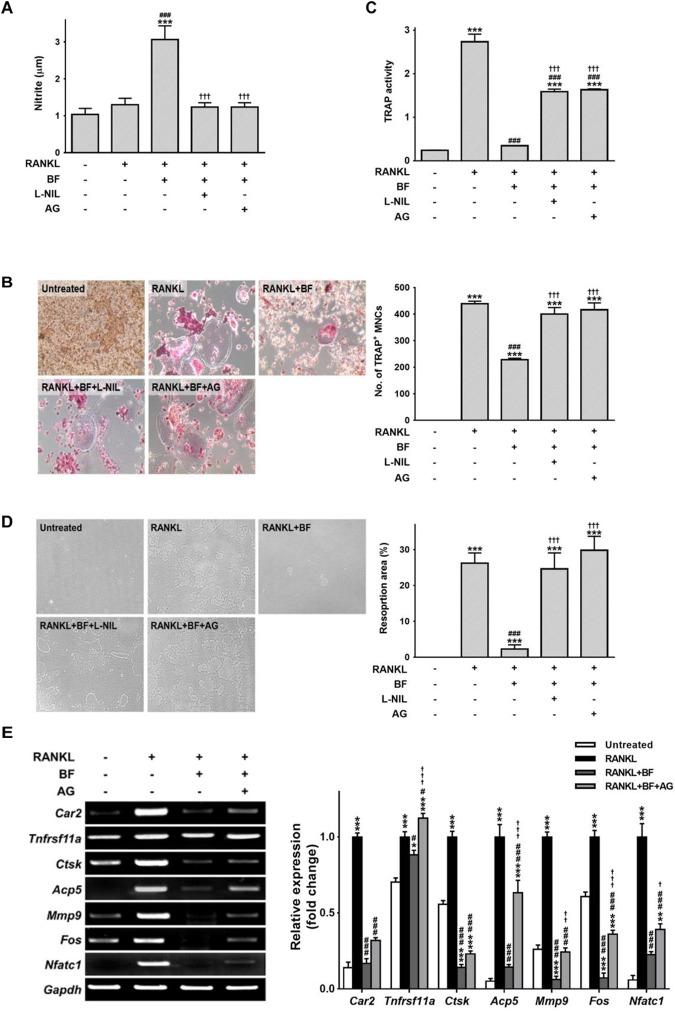
The influence of nitric oxide in the inhibition of osteoclastogenesis by BF extract in RANKL-stimulated RAW 264.7 cells. RAW 264.7 cells were pre-treated with the iNOS-selective inhibitors AG (500 μM) or L-NIL (100 μg/mL) for 60 min and cultured with RANKL (100 ng/mL) in the presence or absence of BF (40 μg/mL) for 5 days. **(A)** The nitrite level in cell supernatants was measured by Griess assay. **(B)** RANKL-induced osteoclast differentiation of RAW 264.7 cells into osteoclasts was assessed by TRAP staining, and TRAP-positive multinucleated cells (TRAP+MNCs; ≥ 3 nuclei) were counted as mature osteoclasts. Original magnification, 100×. **(C)** TRAP activity in the cell supernatants was determined using pNPP as a substrate. **(D)** The osteoclasts derived from RANKL-stimulated RAW 264.7 cells were assessed for osteoclast activity by pit formation assay. The resorption pits were visualized by light microscopy. Original magnification, 100×. The resorption area was measured and expressed as a percentage of the total plate area. **(E)** Osteoclasts derived from RANKL-stimulated RAW 264.7 cells were assayed for mRNA expression of the indicated genes by semi-quantitative RT-PCR. *Car2*, carbonic anhydrase 2; *Tnfrsf11a*, RANK; *Ctsk*, cathepsin K; *Acp5*, TRAP; *Mmp9*, matrix metalloproteinase-9; *Fos*, c-fos osteosarcoma oncogene; *Nfatc1*, nuclear factor of activated T-cells, cytoplasmic, calcineurin-dependent 1; *Gapdh*, glyceraldehyde 3-phosphate dehydrogenase. All data are expressed as means ± SEM. Experiments were performed with at least three independent replicates. ^∗^*P* < 0.05, ^∗∗^*P* < 0.01, ^∗∗∗^*P* < 0.001 versus untreated control; ^#^*P* < 0.05, ^###^*P* < 0.001 versus RANKL alone; ^†††^*P* < 0.001 versus RANKL+BF (40 μg/mL).

**FIGURE 9:**
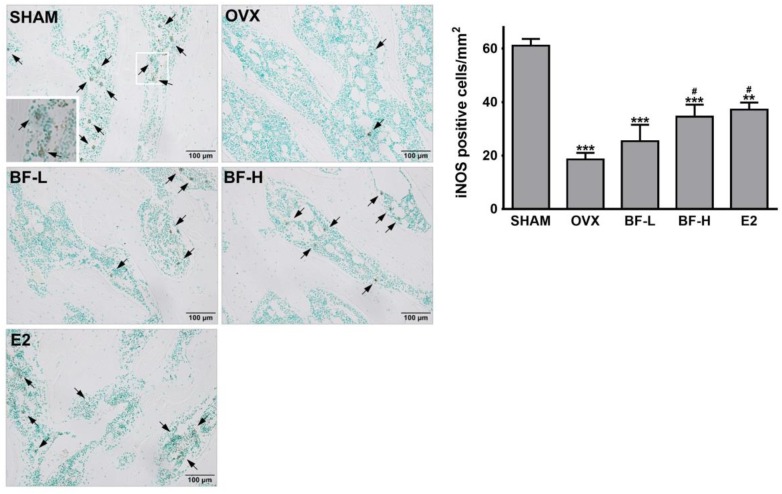
Effects of BF extract on iNOS expression in ovariectomized rats. Proximal femurs were assessed by immunohistochemical staining for iNOS expression. Arrows, iNOS-positive cells. Scale bar = 100 μm. iNOS-positive cells in femoral epiphyses were counted. SHAM, sham-operated control; OVX, untreated ovariectomized rats; BF-L, ovariectomized rats treated with low-dose BF (12 mg/kg); BF-H, ovariectomized rats treated with high-dose BF (120 mg/kg); E2, ovariectomized rats treated with 17β-estradiol (100 μg/kg). Values are expressed as means ± SEM. ^∗∗^*P* < 0.01, ^∗∗∗^*P* < 0.001 versus SHAM group; ^#^*P* < 0.05 versus OVX group.

To further confirm the importance of iNOS induction in the inhibition of osteoclastogenesis by BF, we attempted to inhibit iNOS by using aminoguanidine (AG) or L-*N*-iminoethyl-lysine (L-NIL), two selective competitive inhibitors. AG and L-NIL completely inhibited the augmentation of NO production by BF (Figure 8A), while significantly increasing the number of TRAP-positive OCs and TRAP activity (Figures 8B,C) and bone pit resorption by OCs formed under these conditions (Figure 8D). The mRNA abundance of a series of characteristic OC differentiation markers was also clearly increased by pretreatment with AG (Figure 8E). These results support our general hypothesis that iNOS-derived NO mediates the suppressive effects of BF on RANKL-induced OC formation. Indeed, a dose-dependent increase in iNOS expression was also observed in the proximal femurs of OVX rats treated with BF (Figure 9).

## Discussion

Estrogen deficiency after menopause accelerates an excess of bone resorption over formation, thereby leading to osteoporosis characterized by excessive loss of trabecular bone (Gambacciani and Levancini, 2014). In this study, we demonstrate for the first time that BF may help prevent estrogen deficiency-induced bone loss by inhibiting OC formation and function. We also report that BF may negatively regulate OC differentiation and activation by increasing iNOS/NO signaling.

BF is a medicinal plant with antipyretic, anti-inflammatory, and anti-depressant properties that help relieve menopausal symptoms (Yang and Wang, 2006). These properties suggest the possibility that BF may also have protective effects against menopause-related bone loss, another important health issue for postmenopausal women. We showed that a high dose of BF partially protected against estrogen deficiency-induced bone loss in OVX rats which exhibit most of the characteristics of human postmenopausal osteoporosis (Jee and Yao, 2001). Furthermore, the results on biochemical bone turnover markers showed that BF significantly decreased serum TRAP activity induced by OVX, whereas osteocalcin was not affected by BF treatment in OVX rats. Although TRAP and osteocalcin homeostasis are complex processes, these results indicate that BF probably exerts a more effective action on OCs than on osteoblasts. This was confirmed by the further findings that BF not only attenuated the number of TRAP-positive OCs within the trabecular bone region of proximal femurs in OVX rats *in vivo*, but also almost completely inhibited RANKL-induced osteoclastogenesis *in vitro*. The inhibitory effects of BF on OC differentiation and function were further confirmed by its downregulation of the genes typically expressed by OCs.

c-Fos and NFATc1 are the master transcription factors that regulate the expression of genes associated with RANKL-induced OC differentiation. As expected, BF treatment markedly inhibited c-Fos and NFATc1 at the mRNA and protein levels, indicating that the suppression of c-Fos and NFATc1 induction is an important molecular event in the inhibitory effect of BF on OC formation. c-Fos in particular plays an essential role as a direct transcriptional regulator of NFATc1. c-Fos triggers a transcriptional regulatory cascade by producing NFATc1, thereby activating a number of OC-specific genes such as TRAP and cathepsin K that promote bone resorption (Matsuo et al., 2004). We observed a strong suppression of c-Fos rather than NFATc1 at the protein level following BF treatment. While more direct evidence is needed, these data raise the possibility that BF inhibits the induction of NFATc1 by suppressing c-Fos, which in turn inhibits OC formation.

As BF strongly inhibited c-Fos and NFATc1, it was expected that their upstream signals, the MAPK and NF-κB pathways, would also be decreased by BF treatment. Unexpectedly, BF did not affect RANKL-induced MAPK phosphorylation and NF-κB p65 nuclear translocation but rather appeared to markedly augment MAPK phosphorylation. These findings raise the intriguing possibility that a signaling molecule other than NF-κB or MAPKs is involved in the inhibitory effects of BF on RANKL-induced c-Fos expression. Although clearly essential for OC formation and function, RANKL also triggers negative feedback regulation in OCs that ultimately limits osteoclastic bone resorption. This negative feedback pathway involves IFN-β as a negative feedback controller. In OC precursor cells, IFN-β is endogenously produced in a c-Fos-dependent manner and strongly inhibits OC differentiation by interfering with RANKL-induced c-Fos expression (Takayanagi et al., 2002). This IFN-β-mediated negative feedback regulation occurs without any changes in TRAF6, JNK, p38 MAPK, or NF-κB protein levels (Takayanagi et al., 2002). We showed the upregulation of IFN-β expression by BF treatment during RANKL-induced osteoclastogenesis, suggesting that upregulation of IFN-β may be the mechanism by which BF suppresses RANKL-induced c-Fos expression in OC precursors. However, the upregulation of IFN-β by BF, while statistically significant, was not overwhelming.

Another mechanism relevant to the anti-osteoclastogenic effect of IFN-β is the induction of NO, which has been shown to inhibit OC formation (Zheng et al., 2006). This prompted us to investigate whether BF suppresses RANKL-induced osteoclastogenesis via iNOS/NO signaling. Our results showed that BF treatment markedly increased iNOS expression and subsequent NO release in RANKL-treated RAW 264.7 cells. In addition, increased iNOS expression following BF administration was shown in the proximal femurs of OVX rats. We also showed that the anti-osteoclastogenic effects of BF were abolished by interfering with iNOS/NO signaling with iNOS-selective inhibitors. Although further studies are necessary to rigorously assess the *in vivo* mechanism, the data strongly suggest that BF inhibits RANKL-induced osteoclastogenesis at least in part by enhancing iNOS/NO signaling, an autocrine negative feedback signal. In addition, NO appears to have a biphasic effect on OC formation and function: low concentrations of NO potentiate bone resorption while high concentrations inhibit OC formation and activity (Van’t Hof and Ralston, 2001). This biphasic effect of NO on OC differentiation may also be stage-dependent, as NO initially negatively regulates pre-OC differentiation, but later facilitates the fusion of pre-OCs (Nilforoushan et al., 2009). Therefore, in this study, because it was added at the beginning of stimulation with RANKL, BF appears to inhibit differentiation into pre-OCs and then OC formation and activation with a potent production of NO, indicating that BF may be effective against the development of bone-resorbing OCs. Given that NO has a stage-dependent biphasic effect, BF added with RANKL may be ineffective or exacerbate mature OC formation when added to committed pre-OCs, although further experimentation is needed to verify and validate this hypothesis.

Saikosaponins, the major bioactive constituents of BF, also exhibit potent inhibition of RANKL-induced OC formation *in vitro* (Yu et al., 2013; Shin et al., 2015; Zhou et al., 2015). However, its inhibitory action might be mediated through the inhibition of the NF-κB and MAPK pathways (Zhou et al., 2015), which seems contradictory to our finding that BF augmented the activation of the NF-κB and MAPK pathways, albeit efficiently suppressing OC formation, in RANKL-stimulated RAW 264.7 cells. Thus, it is conceivable that the inhibitory effects of BF extract against RANKL-induced osteoclastogenesis may be elicited by bioactive constituents other than saikosaponins. In addition, saikosaponins, particularly saikosaponin-d, have estrogen-like effects *in vitro* and *in vivo* (Li et al., 2009; Wang et al., 2010). BF did not alter the decrease in uterine weight to body weight ratio in OVX rats; thus, BF does not have estrogenic activity and the effects of BF on bone resorption are not dependent on sex steroids. The implication that BF may be used to attenuate bone loss in postmenopausal women without affecting uterine tissue deserves further investigation. In addition, the effects of BF in this study are on BF are on the bone loss that occurs immediately following ovariectomy. Thus, further investigation would be necessary to determine the ability of BF to restore the bone loss seen post-ovariectomy which would be of important relevance to the treatment of post-menopausal women with established osteopenia and osteoporosis.

In summary, we have shown that a high dose of BF partially prevented estrogen deficiency-induced bone loss with anti-osteoclastogenic activity potentially due to the regulation of the c-Fos/IFN-β/iNOS/NO signaling pathway. BF may provide potential therapeutic benefits for osteoporosis beyond its effects in reducing menopausal discomfort such as hot flashes in pre- and post-menopausal women, without inducing estrogen-like proliferative effects on uterine tissue.

## Author Contributions

MY and YS designed the studies. E-YK and J-HK performed the experiments. MY, E-YK, and J-HK performed data interpretation. MY, E-YK, H-SJ, and YS discussed the results. MY and YS coordinated the studies. MY wrote the manuscript. All authors read and approved the manuscript.

## Conflict of Interest Statement

The authors declare that the research was conducted in the absence of any commercial or financial relationships that could be construed as a potential conflict of interest.
