# Supplementary material for: High Doses of Bupleurum falcatum Partially Prevents Estrogen Deficiency-Induced Bone Loss With Anti-osteoclastogenic Activity Due to Enhanced iNOS/NO Signaling
Source: Front Pharmacol. 2018 Nov 16;9:1314. doi: 10.3389/fphar.2018.01314 (PMC6262412; doi:10.3389/fphar.2018.01314)

**High doses of *Bupleurum falcatum* partially prevents estrogen deficiency-induced bone loss with anti-osteoclastogenic activity due to enhanced iNOS/NO signaling.**

Mijung Yeom^1^, Eun-Young Kim^2^, Jae-Hyun Kim^2^, Hyuk-Sang Jung^2^, Youngjoo Sohn^2^.

^1^Acupuncture and Meridian Science Research Center, College of Korean Medicine, Kyung Hee University, Seoul 130-701, Korea

^2^Department of Anatomy, College of Korean Medicine, Kyung Hee University, Seoul 130-701, Korea

Address for correspondence:

Professor Youngjoo Sohn, OMD and Ph.D

Department of Anatomy,

College of Korean Medicine, Kyung Hee University,

26 Kyungheedae-ro, Dongdaemun-gu, Seoul

130-701, Republic of Korea

E-mail: youngjoos@khu.ac.kr

Table S1. Primer sequences and conditions for RT-PCR

Figure S1. The HPLC chromatograms of BF extract and the reference chemical, saikosaponin A, with detection at 215 nm.

Figure S2. Effects of BF extract on cathepsin K expression in ovariectomized rats.

**Table S**1. Primer sequences and conditions for RT-PCR.

| **Gene** |  | **Primer sequence (5′ → 3′)** | **Product size**  **(bp)** | **Annealing**  **(°C)** |
| --- | --- | --- | --- | --- |
| *Car2* | forward | CTC TCA GGA CAA TGC AGT GCT GA | 411 | 58 |
|  | reverse | ATC CAG GTC ACA CAT TCC AGC A |  |  |
| *Tnfrsf11a* | forward | AAA CCT TGG ACC AAC TGC AC | 377 | 53 |
|  | reverse | ACC ATC TTC TCC TCC CGA GT |  |  |
| *Ctsk* | forward | AGG CGG CTA TAT GAC CAC TG | 403 | 58 |
|  | reverse | CCG AGC CAA GAG AGC ATA TC |  |  |
| *Acp5* | forward | ACT TCC CCA GCC CTT ACT ACC G | 381 | 58 |
|  | reverse | TCA GCA CAT AGC CCA CAC CG |  |  |
| *Fos* | forward | ATG GGC TCT CCT GTC AAC AC | 480 | 58 |
|  | reverse | GGC TGC CAA AAT AAA CTC CA |  |  |
| *Nfatc1* | forward | TGC TCC TCC TCC TGC TGC TC | 480 | 58 |
|  | reverse | CGT CTT CCA CCT CCA CGT CG |  |  |
| *Mmp9* | forward | CGA CTT TTG TGG TCT TCC CC | 258 | 58 |
|  | reverse | TGA AGG TTT GGA ATC GAC CC |  |  |
| *Ifnb1* | forward | CTT CTC CAC CAC AGC CCT CTC | 347 | 58 |
|  | reverse | CCC ACG TCA ATC TTT CCT CTT |  |  |
| *Gapdh* | forward | ACT TTG TCA AGC TCA TTT CC | 267 | 57 |
|  | reverse | TGC AGC GAA CTT TAT TGA TG |  |  |

*Car2*, carbonic anhydrase 2; *Tnfrsf11a*, RANK; *Ctsk*, cathepsin K; *Acp5*, TRAP; *Fos*, c-fos osteosarcoma oncogene; *Nfatc1*, nuclear factor of activated T-cells, cytoplasmic, calcineurin-dependent 1; *Mmp9*, matrix metallopeptidase-9; *Ifnb1*, interferon beta 1; *Gapdh*, glyceraldehyde 3-phosphate dehydrogenase.

Figure S1.


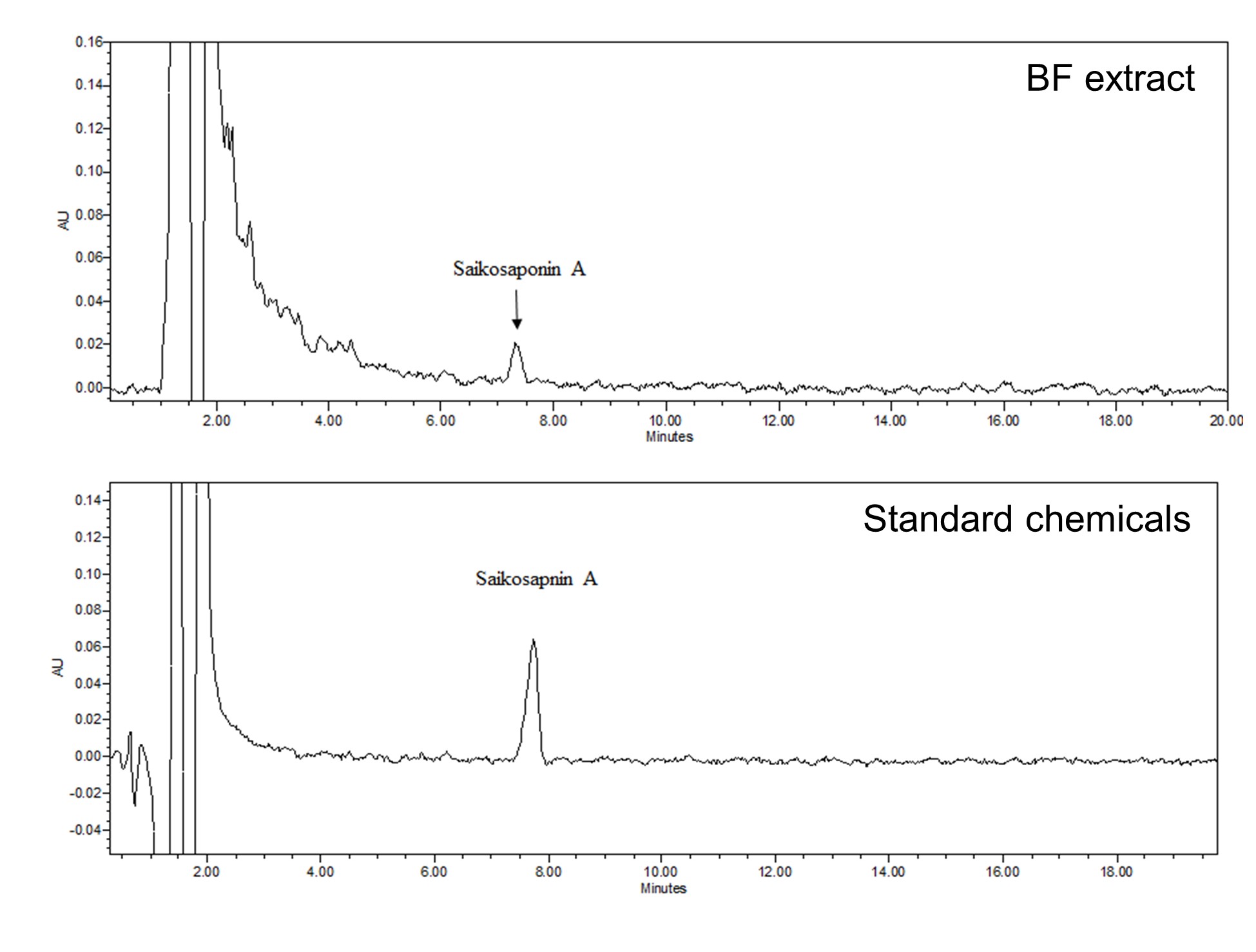


Figure S2.


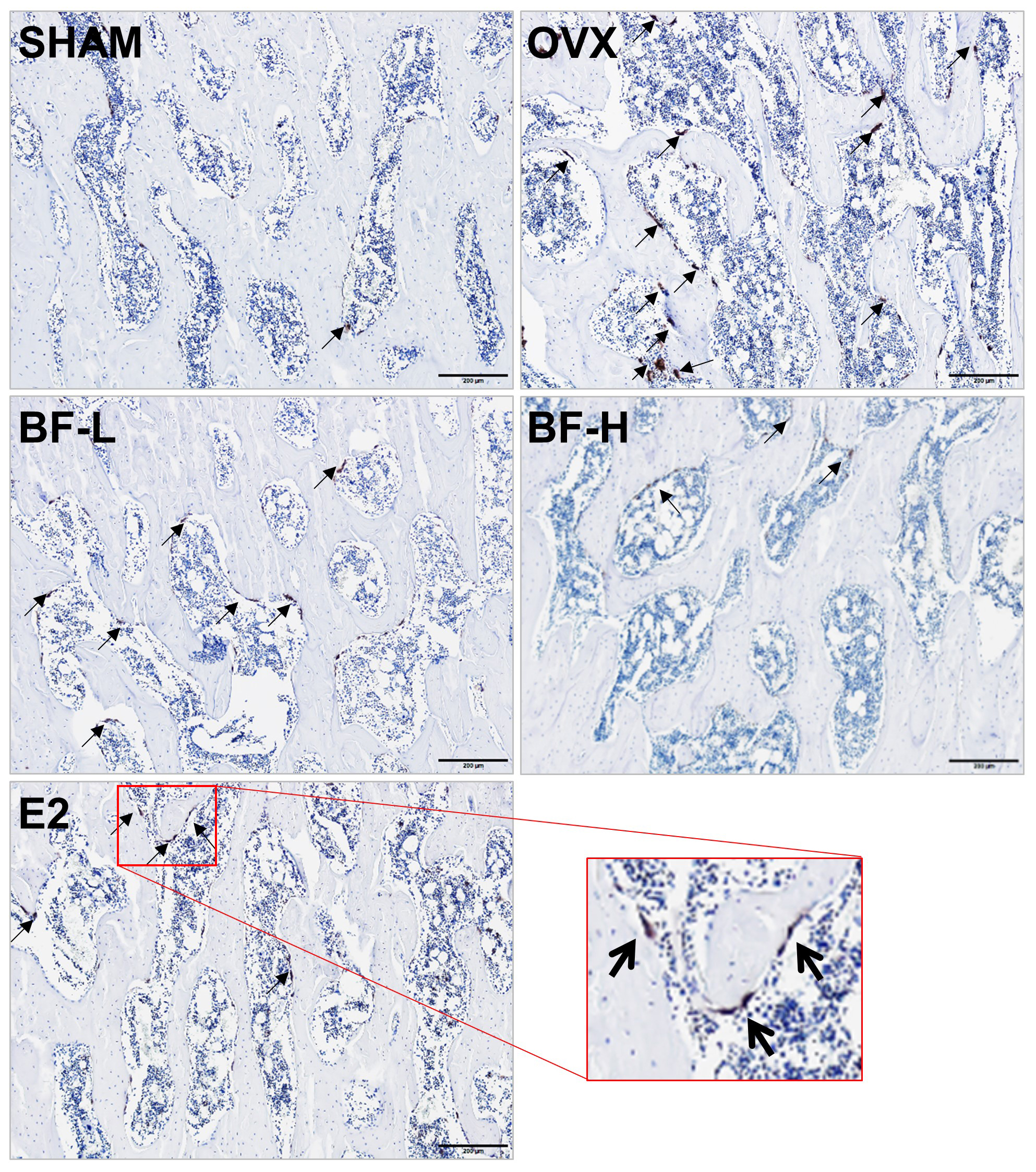

Supplement: Supplementary file 1 [file Data_Sheet_1.doc]
